# Temporal uncertainty in disease diagnosis

**DOI:** 10.1007/s11019-023-10154-y

**Published:** 2023-05-24

**Authors:** Bjørn Hofmann

**Affiliations:** 1 Centre for Medical Ethics, Institute for Health and Society, Faculty of Medicine, PO Box 1130, Oslo, N-0318 Norway; 2 Institute of the Health Sciences, The Norwegian University of Science and Technology (NTNU), Gjøvik, Norway

**Keywords:** Diagnosis, Anamnesis, Prognosis, Uncertainty, Ethics, Epistemology

## Abstract

There is a profound paradox in modern medical knowledge production: The more we know, the more we know that we (still) do not know. Nowhere is this more visible than in diagnostics and early detection of disease. As we identify ever more markers, predictors, precursors, and risk factors of disease ever earlier, we realize that we need knowledge about whether they develop into something experienced by the person and threatening to the person’s health. This study investigates how advancements in science and technology alter one type of uncertainty, i.e., temporal uncertainty of disease diagnosis. As diagnosis is related to anamnesis and prognosis it identifies how uncertainties in all these fields are interconnected. In particular, the study finds that uncertainty in disease diagnosis has become more subject to prognostic uncertainty because diagnosis is more connected to technologically detected indicators and less closely connected to manifest and experienced disease. These temporal uncertainties pose basic epistemological and ethical challenges as they can result in overdiagnosis, overtreatment, unnecessary anxiety and fear, useless and even harmful diagnostic odysseys, as well as vast opportunity costs. The point is not to stop our quest for knowledge about disease but to encourage real diagnostic improvements that help more people in ever better manner as early as possible. To do so, we need to pay careful attention to specific types of temporal uncertainty in modern diagnostics.

## Introduction

Diseases are phenomena that throughout history have been considered to occur in time and space (location) (Evans et al. 2016; Whitbeck 1977; White 1926). The *space* of disease has been studied extensively in the history of medicine where diseases have been identified in body fluids in terms of humoral imbalances (Hippocrates, Galenos), in organs (Giovanni Battista Morgagni, 1682–1771), tissues (Xavier Bichat, 1771–1802), cells (Rudolf Virchow, 1821–1902), biomolecules (genes) and other places (Hofmann 2001, 2017b).

Correspondingly, disease is studied in *time*, e.g., in terms of its temporal extension (lasting in time)(Hofmann 2002). Historically, the temporal aspects of disease have been studied in terms of the ancient concepts of *Chronos* and *Kairos* (Bergmann 2003). *Chronos* has been crucial in studying the chronological development of disease, and *Kairos* has traditionally been a key term for studying the right time for treatment.

In modern (preventive) medicine time has become ever more important, e.g., in terms of detecting and treating disease as early as possible. In particular, a wide range of screening programs have been implemented and early detection has become part of clinical care. Moreover, the development of biomarkers and use of artificial intelligence (machine learning and deep learning) and BigData are envisioned extensively to preempt disease (Ginsburg and Willard 2009; Young 2021).

However, there seems to be a profound paradox in modern medical knowledge production: The more we know about disease development, the more we know about the uncertainties of disease diagnosis, i.e., the more we know that we (still) do not know. Nowhere is this as visible as in early detection of disease. Novel knowledge about predictors, precursors, and risk factors reveals the urgent need for knowledge about whether such indicators develop into something that will be experienced by the person and threatening to the person’s health (Welch et al. 2011). That is, while the vast advances in science and technology have identified a wide range of (bio)markers, signs, signals, findings, red flags, and alarms that are associated with disease, oftentimes we do not know whether they will develop into something that will ever bother the examined, monitored, or tested person, i.e., that will result in pain, dysfunction, or suffering in individuals (Hofmann and Welch 2017).

Hence, while science and technology vastly has expanded our knowledge about temporal aspects of disease (such as disease mechanisms and progression), they also have revealed how much more there is still to know. This increase in *temporal uncertainty* poses basic epistemological and ethical challenges as it can result in overdiagnosis, overtreatment, unnecessary anxiety and fear, useless and even harmful diagnostic odysseys, as well as vast opportunity costs.

Accordingly, the objective of this article is *to investigate the temporal uncertainty that results from advancements in diagnostic science and technology*. The research question is: *how do advancements in science and technology alter temporal uncertainty in disease diagnosis in modern medicine?* As the uncertainties of diagnosis are closely connected to anamnesis and prognosis, relevant aspects of anamnesis and prognosis will be addressed together with the temporal uncertainty of diagnosis.

In order to address the research question, the article will follow four steps. In the first step, the concepts of diagnosis, anamnesis, and prognosis will be briefly defined. Second, basic temporal uncertainties in anamnesis, diagnosis, and prognosis will be investigated. The third step will investigate how disruptive science and technology have altered basic temporal elements of medical knowledge production and application and how this can enhance or introduce new types of uncertainties. The fourth step is based on the analysis in the previous steps and argues that advances in science and technology have (over time) resulted in a closer connection between diagnosis and prognosis and increased our prognostic uncertainty for a wide range of diagnosed conditions, weakening the epistemological standing of diagnoses.

## Anamnesis, diagnosis, and prognosis

*Anamnesis* is the medical history of a patient. The word stems from new Latin and Greek (anámnēsis “remembrance,” equivalent to ana(mi)mnḗ(skein) “to remember” or “to call to mind”). The anamnesis presents the development of disease as experienced by the patient, i.e., the development of illness. Based on the anamnesis health professionals aim to reach a diagnosis of a disease and a treatment plan (Sadegh-Zadeh 2012). Hence, anamnesis tracks the disease backwards in time in order to identify it in the present (diagnosis), to predict its temporal course (prognosis), and to influence the progression of the disease, where possible (treatment).

The term *diagnosis* stems from New Latin and from Greek (diágnōsis, “a distinguishing, means or power of discernment”). The meaning has changed throughout history, but one traditional meaning seems to be “discerning by knowledge” (Liddell et al. 2011). Diagnosis has been considered to be categorical or conjectural statement about specific conditions in the world, a label (idiom) (Sadegh-Zadeh 2012), and a value-laden (Stempsey 1999) social construct (Brown 1995). Moreover, it has been used as a classificatory category (Jutel 2011) and the name of a process (Blaxter 1978) and, relatedly, as nosographic and pathophysiological types (Chiffi and Zanotti 2017). According to one definition diagnosis is “a hypothesis about the nature of a patient’s illness, one that is derived from observation derived by the use of inference” (Kassirer 1989). In the context of this study, the term diagnosis both refers to a (disease) label given at a specific time and the temporal process of giving such a label.

*Prognosis* is ”[t]he likely outcome or course of a disease; the chance of recovery or recurrence.”(National Cancer Institute 2015) The term stems from Late Latin and Greek prógnōsis “foreknowledge.” As such it is knowledge about the progression of a disease in time, or more precisely the prediction of the progression of a disease (or condition) in time. Moreover, prognosis involves two types of predictions, i.e., what will happen with and without intervention (Sadegh-Zadeh 2012), and as such is imbued with uncertainty (Chiffi and Zanotti 2017).

Hence, while anamnesis looks *backward* in time, prognosis looks *forward*. Anamnesis points towards a diagnosis at a given time. Nonetheless, diagnosis is not a-temporal as it develops through a process and points to a prognosis (depending on the knowledge about the disease).

### Temporal uncertainties in anamnesis, diagnosis, and prognosis

Paul Han and colleagues have mapped a wide range of sources of uncertainty (probability, ambiguity, complexity), uncertainty issues (scientific, practical, personal), and identified loci of uncertainty (patient, clinician, and researcher) (Han et al. 2011). A recent systematic review by Bhise and colleagues uncovers a rich literature on diagnostic uncertainty and concludes that “diagnostic uncertainty” lacks a clear definition (Bhise et al. 2018). Correspondingly, a wide range of errors (causing uncertainty) have been identified in diagnostics and prognostics (Balogh, Miller, Ball, National Academies of Sciences, & Medicine, 2015; Graber 2013; Graber et al. 2005; Newman-Toker and Pronovost 2009; Norman and Eva 2010; Norman et al. 2017; Pinto and Brunese 2010; Smith et al. 2013). This study adds to the existing literature in looking more narrowly at the temporal uncertainty involved in disease diagnosis.

In the *anamnesis*, the main temporal uncertainty is of a historical kind, e.g., related to remembering past events, such as symptoms, health-related events, medications etc. Accordingly, there may be recollection error, confirmation bias, lack of attention, or lack of knowledge (health illiteracy). Additionally, there is uncertainty with respect to whether the various identified previous health-related issues are relevant for the present situation. Hence, the temporal uncertainty of anamnesis stem from *ignorance* (of important events, factors, and preconditions) and uncertainty of the *relevance* (of such factors).

The (temporal) uncertainties from anamnesis may influence diagnostics, e.g., with respect to deciding which examinations to make, which tests to take, and to estimate the pre-test and post-test probability of a specific disease (Hofmann and Lysdahl 2021). The uncertainty depends on the outcomes of each diagnostic step and varies throughout the diagnostic process (Seely 2013). For example, each step in diagnostic imaging has its own sources of uncertainty (Hofmann and Lysdahl 2021). Hence, “diagnostic uncertainty is dynamic and changes with time.” (Bhise et al. 2018) The uncertainties in the process of *diagnosis* are related to the accuracy of the test, i.e., whether the test misses cases of disease (due to low sensitivity) and diagnoses cases of non-disease (due to low specificity). Correspondingly, positive and negative test results may be wrong (due to low predictive values). Moreover, diseases may have undiscovered variants (due to *ignorance*) and their signs have unknown probability distributions (*fundamental uncertainty*) or be defined ambiguously (*indeterminacy*) (Hofmann and Lysdahl 2021; Wynne 1980, 1992).

In sum, uncertainty varies throughout the diagnostic process depending on uncertainties about the test characteristics, test context, significance of symptoms, signs, findings, and test results. Moreover, even for categorical diagnosis, where the question is whether the person’s condition falls under the concept of a specific diagnosis or not, there is temporal uncertainty, for example for asymptomatic and presymptomatic conditions there is uncertainty with respect to whether the person will develop symptoms and manifest disease.

In *prognosis* there is temporal uncertainty with respect to how the diagnosed condition (mostly a disease) will progress. This *progression uncertainty* stems from the fact that (instances of) diseases develop differently. Some may develop rapidly, others slowly, some may stop progressing, and some even regress. Moreover, diagnoses are not the same as diseases. As clearly stated in the ICD (from ICD-10 in 1989 and onwards) many diagnoses are not labels of diseases, but of health-related problems and risk-factors. Moreover, the term “diagnosis” is used for discerning conditions that are explicitly not considered to be diseases. One example of this is when professionals are “diagnosing pregnancy” (Cohen and Teal 2022). Hence, one type of temporal uncertainty is whether what is identified (and labelled) with a diagnosis will (ever) progress to something that is recognized as a (manifest) disease and experienced by the person as pain, dysfunction, and/or suffering (Hofmann, 2017a; Hofmann 2019a).

Thus, three types of temporal uncertainties can be identified in the process from anamnesis to prognosis. First, there is uncertainty with respect to the recollection and relevance in the anamnesis. Second, there are uncertainties in each step of the diagnostic process. Thirdly, partly based on uncertainties in anamnesis and diagnosis and partly on limited knowledge and evidence, there is uncertainty with respect to prognosis, i.e., whether findings will develop into manifest and experienced disease. Table 1 provides an overview of various types of uncertainty related to anamnesis, diagnosis, and prognosis.

**Table 1:** Uncertainties related to anamnesis, diagnosis, and prognosis. For details, see (Hofmann and Lysdahl 2021)

	Anamnesis	Diagnosis	Prognosis
Temporal relevance	Backward-looking	Based on past and expecting future events Process	Forward-looking
Types of uncertainty	Recollection Relevance	Uncertainties in each step of the diagnostic process: • Risk • Fundamental uncertainty • Ignorance • Indeterminacy	Prognostic uncertainty: • Progression uncertainty • Development uncertainty
Sources of uncertainty	Recollection error Lack of knowledge, lack of attention	Lack of information over time due to: Diagnostic accuracy Inappropriate test Unknown probability distribution Unknown events Ambiguity, complexity	Lack of knowledge (about mechanisms and development) Ignorance
Type of temporal uncertainty	About the meaning of symptoms and reported experiences	About the meaning of signs, findings, and test results	About whether findings will develop into something relevant for the person’s experience of pain, dysfunction, and/or suffering

## Disruptive technologies

As technologies have changed medicine since the invention of the uroscope and the stethoscope (1816) (Keers 1981), a wide range of novel technologies have been claimed to be disruptive to medical practice and knowledge production. Artificial intelligence (AI) including Machine learning (ML), BigData, come together with precision medicine (Ho et al. 2020; Nayarisseri et al. 2021; Santosh and Gaur 2021) and are envisioned to totally change medicine. The same goes for genetic screening (and gene editing).

With BigData analysis of large health data as well as behavioral data by various types of AI/ML it is expected that we will get new and unforeseen information about people’s health. This will provide crucial input for prevention and early intervention, but also result in false alarms and unnecessary diagnostic and therapeutic odysseys (Hofmann and Welch 2017). The point here is not to go into the details of how these technologies change medicine, but to use them as examples of how they can influence the temporal uncertainty in disease diagnosis and how this relates to uncertainties in anamnesis and prognosis.

### Technology reducing and enhancing temporal uncertainty

As acknowledged, the role of science and technology is ambiguous and ambivalent. Science and technology have made diseases more fine-grained, knowledge of disease mechanisms and etiology ever more advanced, and the gathering, evaluation, and exchange of evidence better, resulting in reduced temporal uncertainty and improved diagnosis and prognosis.

**Fig. 1:**
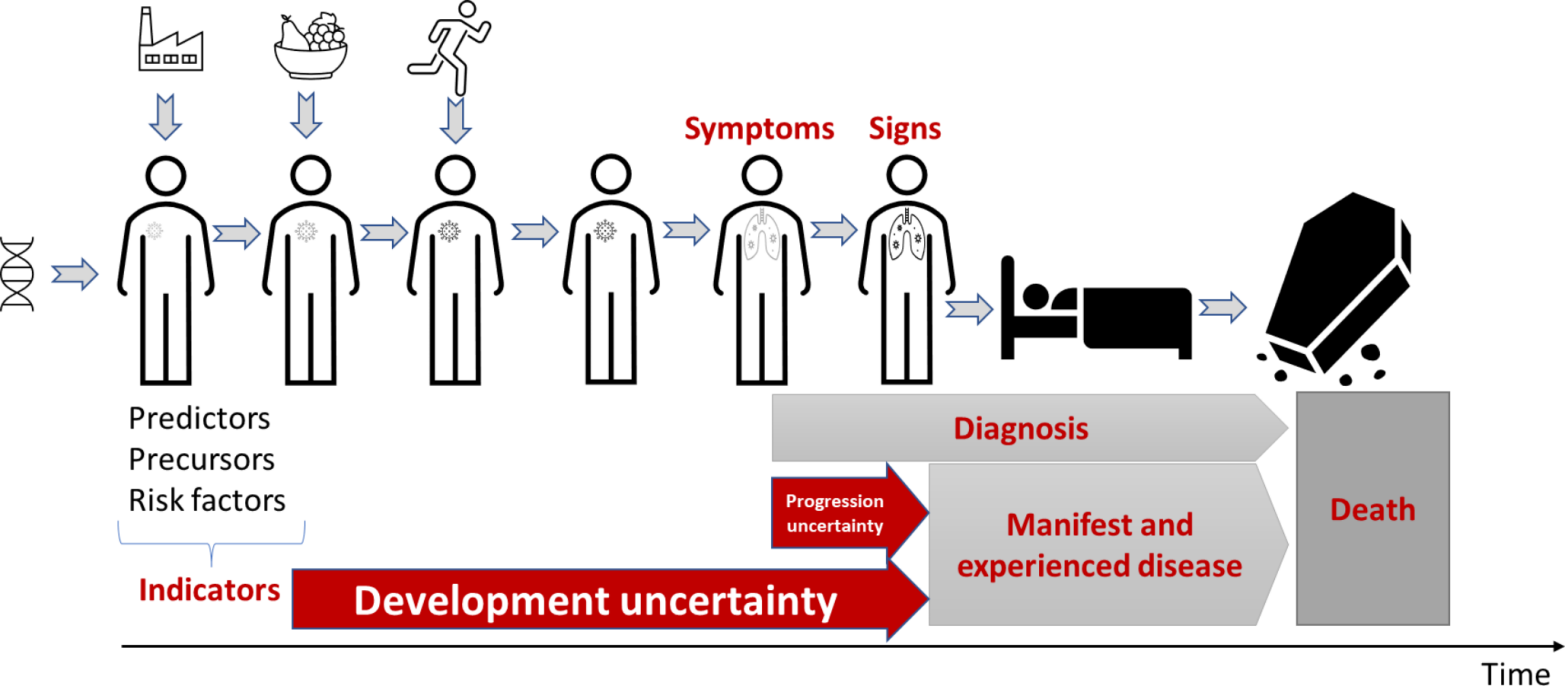
The relationship between progression uncertainty and development uncertainty, who together make up prognostic uncertainty. Symbols above person indicate environmental and lifestyle factors (food and exercise). Symbol left of person indicates genetic makeup. Symbols inside person indicates biomedical changes that can be detected (indicators) and that may develop to symptoms, signs, and manifest and experienced disease. For more details, see (Hofmann 2019a)

At the same time, science and technology have identified a wide range of predictors, risk factors, precursors, preconditions, and biomarkers of disease (here collectively called “indicators”) where we do not know whether they will temporarily evolve to manifest disease and suffering (if they were not detected or treated). This has generated a new type of temporal uncertainty: *development uncertainty*. Development uncertainty is uncertainty of whether what is detected by an indicator will develop into anything that is experienced by the person, such as pain, dysfunction, suffering, manifest disease, or death (Hofmann 2019b). Development uncertainty is different from *progression uncertainty*, which is an uncertainty about how a condition diagnosed (based on symptoms and signs) will progress to manifest and experienced disease. Together development and progression uncertainty make up *prognostic uncertainty*. Figure 1 illustrates the relationship between progression uncertainty and development uncertainty.

When science and technology have revealed clear connections between various indicators and manifest disease or verified disease mechanisms, this has contributed to reducing progression uncertainty and development uncertainty. However, as the connections are rarely clear (or verified) and the mechanisms at best oftentimes are weak, the opposite occurs: prognostic uncertainty increases. In general, the number of biomarkers has increased rapidly, but their connection to what matters to people (pain, dysfunction, and suffering) seems ever weaker.

One reason for this increase in prognostic uncertainty is that advances in science and technology have moved diagnosis ever earlier in time (increasing temporal uncertainty) and away from what matters to people (e.g., reduced the phenomenological relevance)(Aronowitz 2009; Leder and Jacobson 2014). In the latter case, science and technology has contributed to an increased (epistemological and ontological) gap between diagnosis and manifest disease. This development is characterized by two effects. First, diagnosis have become less labels of diseases and more characteristics of bodily or mental phenomena of relevance to health. As we are able to technologically detect and define phenomena that are considered to be relevant for peoples’ health, these are prone to become diagnoses. This happens in various types of early diagnosis, such as in health surveillance and prevention (screening), but also in clinical practice in ordinary testing (including incidentalomas). Hypertension and hyperglycemia are but two examples. As expressed by Aldous Huxley: “Modern medicine has made such tremendous progress, that there is hardly a healthy human left.” (Yudkin and Montori 2014).

Second, the distinction between diagnosis and prognosis has become blurred as progression and development uncertainty have become relevant for diagnosis. That is, in addition to the temporal uncertainties of the diagnosis process, the temporal uncertainty of whether indicators will develop into something relevant for persons’ health makes diagnosis even more subject to temporal uncertainty. More precisely, in addition to the uncertainty at each temporal step of the diagnostic process, uncertainty with respect to whether what the diagnosis indicates will occur is added.

**Fig. 2:**
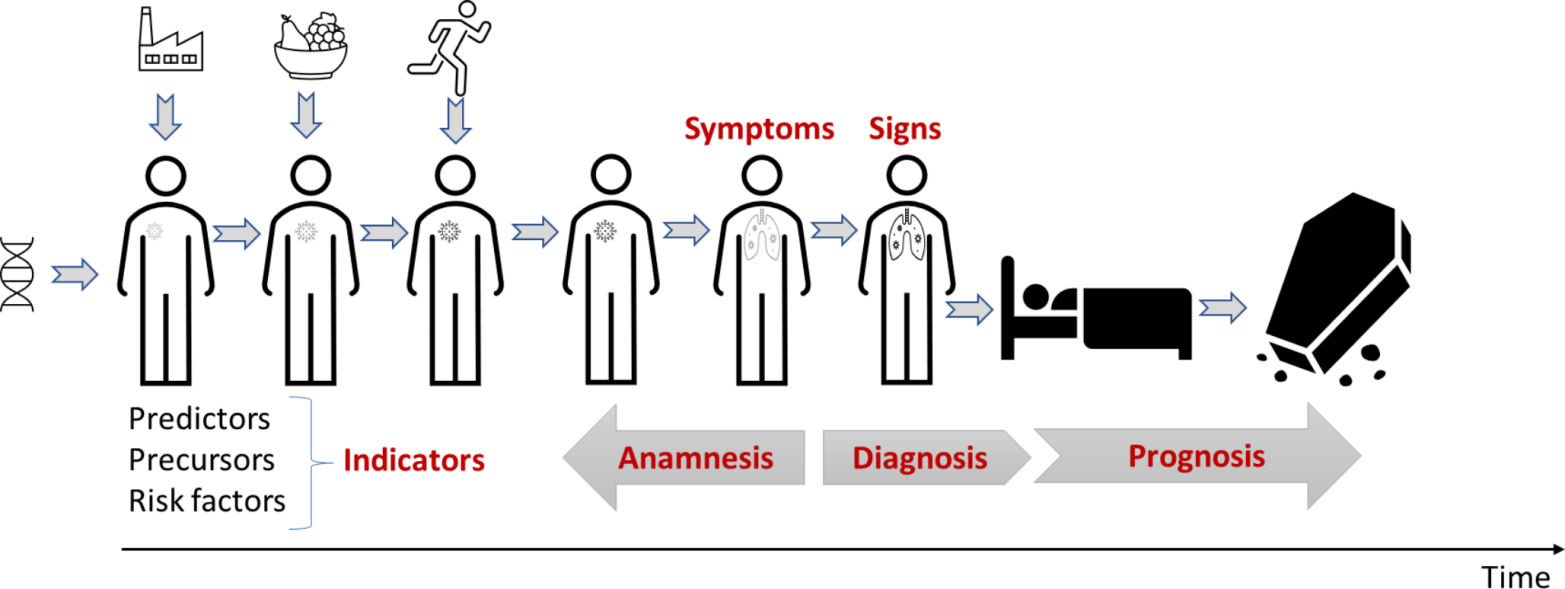
Idealized disease trajectory indicating the relationship between anamnesis, diagnosis, and prognoisis

**Fig. 3:**
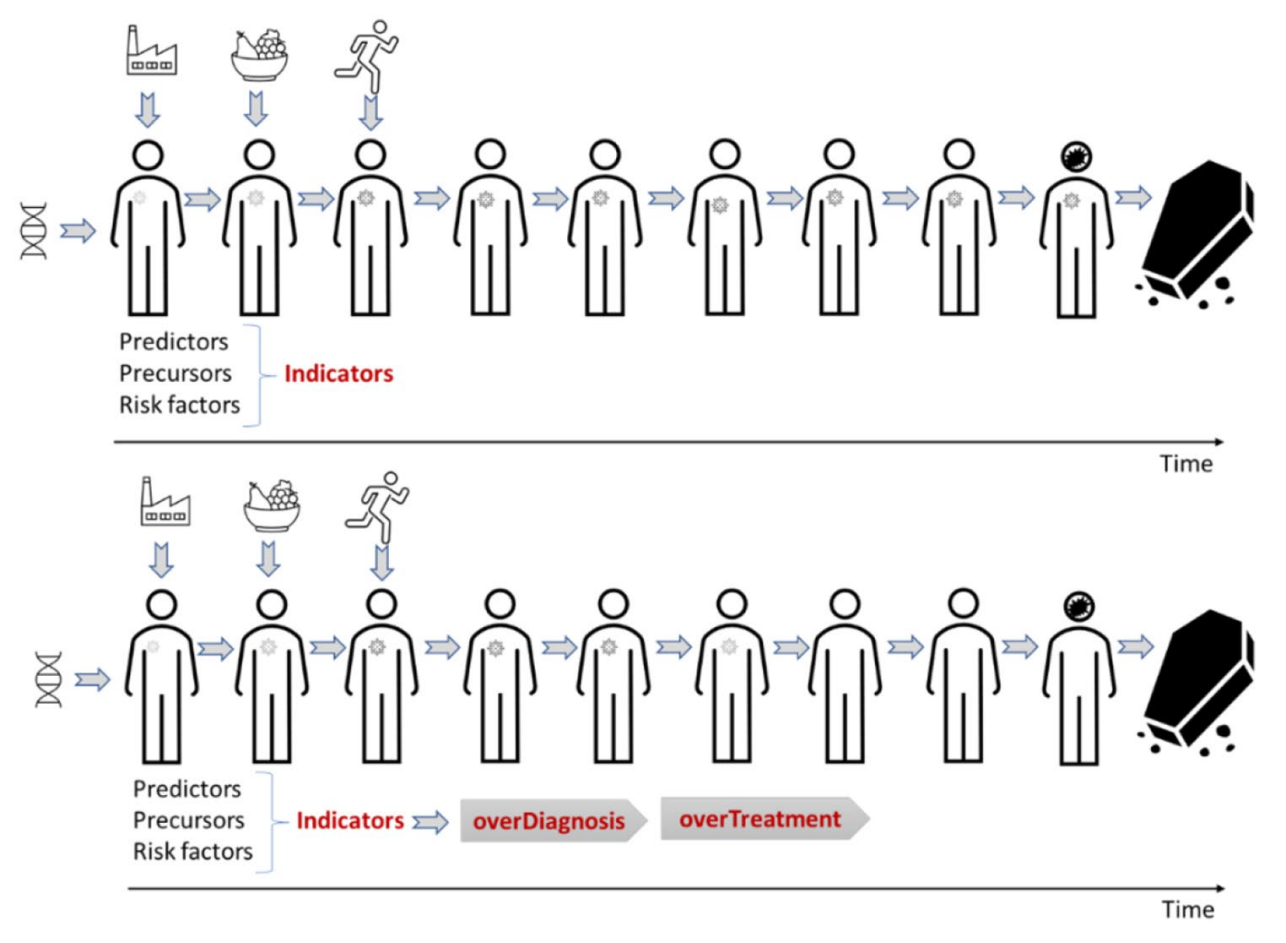
Early detection of indicators of conditions that can develop to disease, but that do not necessarily do so. Upper figure is when left untreated, where the person dies from other causes. Lower figure is when the person is diagnosed and treated, i.e., overdiagnosed and overtreated. In this case there may also be side effects from diagnostics and treatment in addition to experienced fear and anxiety from unnecessary diagnosis and pain and suffering from unnecessary procedures

Figure 2 illustrates the idealized disease trajectory, where indicators may detect a disease process early. However, they may also result in unnecessary anxiety, diagnostic odysseys, overdiagnosis, and overtreatment, as indicated in Fig. 3.

### Diagnosis and prognosis over time

Three important lessons can be drawn from this. First, technology is a game-changer. At the same time as it makes it possible to increase our knowledge about disease mechanisms and developments, and thereby can reduce (prognostic) uncertainty, technology can increase this uncertainty by detecting indicators of conditions that *can* develop into disease ever earlier, but without knowledge of whether they actually *will* do so. While early identification certainly can save lives, it can also result in overdiagnosis, overtreatment, unnecessary anxiety, and fear, as well as vast opportunity costs (Welch 2015; Welch et al. 2011). The reason for this is that the earlier and the more indicators of disease technology identifies, the more uncertain it may be whether they are of any relevance.

Second, the traditional relationship between anamnesis, diagnosis, and prognosis is changed. Anamnesis may (partly) be replaced by algorithm-based alarms and tests for indicators. Where the validity of pretest probability increases, this is certainly a good thing. However, this may not be the case as validation of indicators, such as biomarkers, is not an easy task (Bossuyt 2011; Mohanty et al. 2021; Rodríguez et al. 2021). Moreover, diagnosis may be made based on indicators, and less on anamnesis, symptoms, and traditional signs. This may increase the gap between diagnosis and what matters to people, i.e., pain, dysfunction, and suffering. We get what Aronowitz has called “symptom-less and sign-less disease” (Aronowitz 2009).

Hence, it can be argued that indicator-based diagnosis increases *progression uncertainty* by conflating it with *development uncertainty*. In sum, it increases the temporal uncertainty with respect to whether you would ever have manifest and experienced disease resulting from the identified condition during your lifetime if it were not detected.

Third and more specifically, the relationship between diagnosis and prognosis is changed. While diagnosis may be conceived of as a label of a temporal state of affairs, it now is clear that it is (even) more influenced by potential future events, i.e., by prognosis. Indicators of conditions that can develop to manifest and experienced disease are considered as diagnosis when they can progress into something clinically relevant, including treatment-responsiveness (Armstrong 2009). Hence, potential prognoses strongly influence diagnoses.

**Fig. 4:**
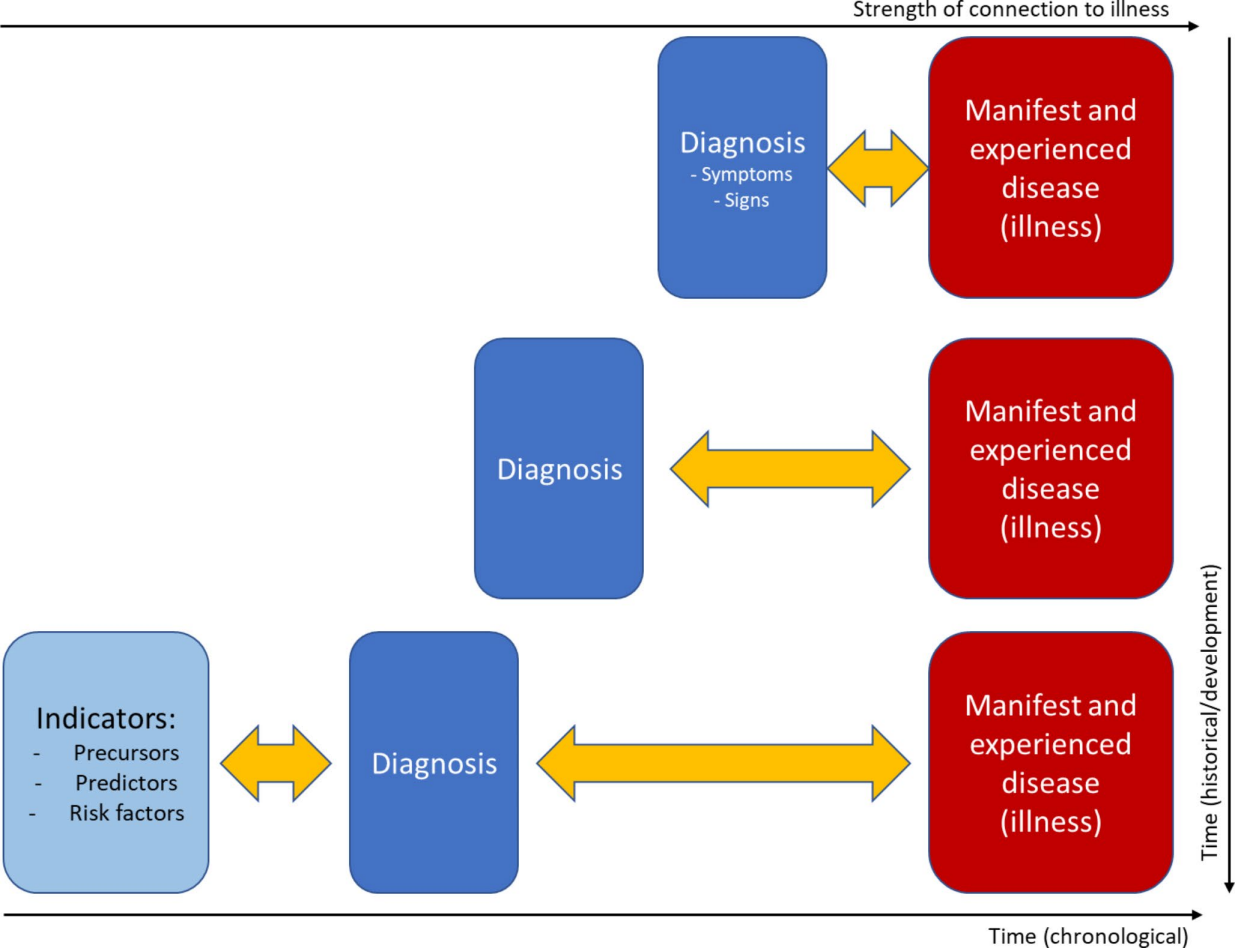
Illustration of how diagnosis has altered its connectedness with manifest and experienced disease (illness) over time as indicators of conditions that may develop into disease are detected ever more early. Lower X-axis is chronological time (from detected indicators to diagnosis and manifest and experienced disease). Right (inverted) Y-axis is historical development over time, where there is an increased (epistemological and temporal) gap between diagnosis and manifest and experienced disease. The latter is illustrated by the upper X-axis indicating the strength of the relationship between diagnosis and experienced disease, i.e., illness

This effect could counterbalance the increasing gap between diagnosis and manifest and experienced disease. However, as we tend to be risk aversive, and subject to progress bias (Hofmann 2019b, 2020), this does not seem to appear. On the contrary, indicators of conditions that have any (weak) connections to manifest disease and illness are prone to become diagnoses. Figure 4 illustrates how the relationship between diagnosis and manifest and experienced disease has changed over time with the introduction of advanced technologies facilitating ever earlier detection of conditions that potentially may develop into disease.

## Discussion

In this article I have argued that science and technology play paradoxical and ambivalent roles: they can both reduce and increase temporal uncertainty. Technologies, such as biomarkers, BigData, AI, ML, and various kinds of precision medicine can come to change the relationship between anamnesis, diagnosis, and prognosis. In particular, diagnoses are less closely connected to manifest and experienced disease resulting in *progression uncertainty*. Additionally, a host of new indicators are even less closely connected to manifest and experienced disease, resulting in increased *development uncertainty*. Together, science and technology have increased *prognostic uncertainty*, which is a crucial type of temporal uncertainty resulting in epistemological and ethical challenges related to phenomena such as overdiagnosis, overtreatment, unnecessary anxiety and fear, useless and even harmful diagnostic odysseys, as well as vast opportunity costs. In doing so, diagnosis has inherited temporal uncertainty from prognosis, i.e., whether what has been diagnosed ever will become manifest and experienced disease (and how).

The claim that science and technology influence the relationship between prognosis and diagnosis may be criticized from at least two points. First, it can be argued that diagnosis has been and is a label of given condition at a specific time. It is categorical and a-temporal (Blaxter 1978; Sadegh-Zadeh 2012), and not influenced by prognosis or other temporal matters, such as anamnesis. I think it is hard to justify such a claim as diagnoses have a moral purpose: to help people. The urgency to help depends on the severity, which depends on the prognosis. If the diagnosed condition will not result in something painful or harmful, it will not qualify as a diagnosis.

Second, it can be argued that diagnoses have been influenced by prognosis throughout history, so the relationship between diagnosis and prognosis has not changed (substantially). If a phenomenon, e.g., a physiological or mental condition, was known to progress to disease, it got a diagnosis label. Ductal carcinoma in situ may serve as an example (Esserman et al. 2014), where a condition (cell changes) is labelled after a potential disease (cancer).

However, as has become ever more evident, the influence of prognosis on diagnosis has increased (prognostic) uncertainty as technologies have been able to identify ever more indicators ever less connected to manifest and experienced disease. Medical doctors, researchers, and philosophers therefore discuss non-harmful diseases (Rogers and Walker 2017). The problem with this is that we remove disease labelling from what matters to people, i.e., pain, dysfunction, and suffering (Hofmann 2019a; Rogers and Mintzker 2016). Accordingly, we reduce our moral obligations towards persons with diagnoses.

Additionally, it may be argued that diagnoses are not necessarily categorical concepts or processes but can be hypotheses of conditions persons may have (conjectural diagnosis) (Sadegh-Zadeh 2012). That is correct, but the hypotheses are temporal and will (by differential diagnoses, by testing treatments, or “test of time”) be corrected or reach a conclusion. In particular, in the “test of time”, where the diagnostic strategy is to test and retest a patient over time to reduce uncertainty in populations of low prevalence (Almond and Summerton 2009; Irving and Holden 2013) the uncertainties are as described above (Table 1).

Besides, diagnostics is used in a wide range of settings, such as for confirmation/exclusion, triage, monitoring, prognosis, screening (Bolboacă 2019), and check-ups and it can be argued that the temporal uncertainty is not the same for all of these. As I have acknowledged, development and progression uncertainty are most prevalent in screening, but are also highly relevant in other diagnostic settings.

Furthermore, I have applied a broad and implicit definition of temporal uncertainty, as this study has been explorative. Nonetheless, three conceptions of timing have appeared throughout the study. Firstly, I have identified uncertainty with respect to the past (recollection, anamnesis). Second, there is uncertainty in the various steps of the diagnostic process in time (present, diagnosis). Third, I have identified two types of uncertainty with respect to the future (progression, development).

Moreover, as pointed out by many scholars, diagnoses are malleable and pragmatically organized (Töpfer and Wiesing 2005; Wieland 2013) and influenced by a wide range of drivers. I fully agree with this. My point here is that the moral appeal to help people stems from the negative experience of manifest and experienced disease in terms of pain, dysfunction, or suffering (Mayerfeld 1999).

Clearly, diagnosis always seems to have been influenced by prognosis (Bolboacă 2019), as there has been a “dia-prognostic” connection, and there is a whole new are of “theranostics.” Acknowledging this, the point here is to provide a more conceptual analysis of the relationship between diagnosis and prognosis, especially related the vast advances in science and technology. In particular, I have tried to show how the temporal uncertainties of prognostic has added temporal uncertainties to diagnostics.

Relatedly, it may be argued that this study is both about anamnesis and prognosis, and that these concepts should be part of the title. However, the reason these concepts are part of the study is because of their relation to (the temporal uncertainty of) diagnosis. Thorrough analyses of the uncertanty of anamnesis and prognoses warrant more in-depth work than can be fitted in one article.

It may also be maintained that this study does not contain any extensive elaboration of science and technologies or any sophisticated conception of disruption (Topol 2012). I fully agree with this as it has not been the purpose to provide this, but rather to use recent advances in science and technology as examples.

The concepts “development uncertainty” and “progression uncertainty” are not new (Hofmann 2019a). However, in this article they are set in the context of temporal uncertainty in diagnostics and in relation to temporal uncertainty in prognosis. Moreover, much more can be said about the various aspects of temporal uncertainty. The same goes for ignorance (Whooley and Barker 2021). However, this article is limited to temporal uncertainty related to emerging diagnostic technologies in general.

Correspondingly, very much more can be said about the concept of “manifest and experienced disease” than is possible in this article. While manifest disease is the “gold standard” of disease diagnosis, experienced disease (illness) is arguably the “diamond standard” (Hofmann 2019a), i.e., what matters most to people. The shift from these standards to indicators (“aluminum standard”) represents “an epistemic ignorance toward the lived experience of illness” (Whooley and Barker 2021) and a breach with the ethos of medicine (Hofmann 2019c). Correspondingly, much more could be said about the relationship between disease and diagnosis, but this has been well covered elsewhere (Aronowitz 2009; Copeland 1977; Copp et al. 2017; Engelhardt Jr 1985; Jutel 2011; Moynihan 2011, 2013; Nickel et al. 2017; Risør 2016; Stempsey 1999; Tresker 2020; Vitor Pordeus and Rosenberg 2017).

Given the vast attention to health informatics, one can ask whether artificial intelligence (AI), machine learning (ML), together with BigData will solve the problems with (prognostic) uncertainty. Given the massive hype of such technologies (Bossuyt 2011; Fox and Do 2013; Frohlich et al. 2018; Goldfield 2014; Labrique et al. 2013; Mazzanti et al. 2018; Mohanty et al. 2021), this is a highly relevant question. For example, it has been argued that AI can re-humanize medicine (Topol 2019). However, problems with data quality, validation, explainability, replicability, and transferability indicates that the solutions are yet not at hand. It is not clear how the technology that generates the problem has the resources to solve it.

Despite extensive efforts to reduce (diagnostic) uncertainty, uncertainty appears to prevail (Whooley and Barker 2021) and temporal uncertainty is increasing. The saying that “the more we know, the more we know that we do not know” persists. “Absolute certainty in diagnosis is unattainable, no matter how much information we gather, how many observations we make, or how many tests we perform.” (Kassirer 1989) There may of course be many ways to conceptualize the ambiguous role of science and technology in medical evidence production. One way to address the problem that science and technology at the same time can reduce and increase uncertainty is to differentiate between data, information, knowledge, evidence, and wisdom (Car et al. 2019). While we may get vast amounts of data and information, they do not necessarily provide knowledge and evidence to reduce uncertainty. Even more, they do not increase our wisdom. As long as we do not know what the information items mean for people’s experienced life (pain, dysfunction, suffering), they are of little value.

Another implication of this study is that while the concept of *kairos* has mostly been discussed in therapy, e.g., in terms of finding the right time to treat the patient, the temporal uncertainty of diagnosis indicates that there is also a *kairos of diagnosis*. Both with respect to finding the right time of taking a diagnostic test and of making a diagnosis.

As already alluded to, this may also have implications for our moral duties with respect to people with diagnoses and for medical taxonomy and nosololgy. Classifying conditions based on indicators with great temporal (prognostic) uncertainty may not make much sense, as getting diagnoses may be more harmful than helpful.

## Conclusion

This study has identified temporal uncertainties in disease diagnosis and related this to uncertainties in the diagnostic process from anamnesis to prognosis. First, there is uncertainty with respect to the recollection and relevance in the anamnesis. Second, there are uncertainties in each specific step of the diagnostic process. Thirdly, there is uncertainty with respect to prognosis, i.e., whether findings will develop into manifest and experienced disease. Over time diagnosis has become more subject to prognostic uncertainty. This is because diagnosis has become (more connected to indicators that are) less closely connected to manifest and experienced disease.

While science and technology have increased our knowledge of disease mechanisms and progression, they have also enlarged our uncertainty about whether (and how) the diagnosed conditions progress to manifest and experienced disease, i.e., *progression uncertainty*. Moreover, the vast increase in the number of indicators have come together with the tendency to connect them to diagnoses, generating *development uncertainty*. We have many more indicators of disease, but we are ever more uncertain whether what they indicate develops to something that is relevant to people’s health. Hence, together advances in science and technology have (over time) increased our *prognostic uncertainty* for a wide range of diagnosed conditions, weakening the epistemological standing of diagnoses. This increase in temporal uncertainty poses basic epistemological and ethical challenges as it can result in overdiagnosis, overtreatment, unnecessary anxiety and fear, useless and even harmful diagnostic odysseys, as well as vast opportunity costs. The point is certainly not to stop our quest for knowledge about disease, including knowledge on diagnosis (anamnesis and prognosis). We need such knowledge to improve diagnostic classification and to be able to help more people in ever better manner as early as possible. However, in order to obtain this, we need to pay careful attention to temporal uncertainty in the dynamics of anamnesis, diagnosis, prognosis, and disease.

## References

[CR1] Almond SC, Summerton N (2009). Test of time. Bmj.

[CR79] Armstrong D. 2019. Diagnosis: From classification to prediction. *Social science and medicine* 237:112444. 10.1016/j.socscimed.2019.112444. 10.1016/j.socscimed.2019.112444 31374408

[CR2] Aronowitz RA (2009). The converged experience of risk and disease. Milbank Quarterly.

[CR3] Balogh, E. P., B. T. Miller, J. R. Ball, National Academies of Sciences, E., & Medicine. 2015. Overview of diagnostic error in health care. In *Improving Diagnosis in Health Care*: National Academies Press (US).

[CR4] Bergmann G (2003). [Time–the 4th dimision in medicine and psychotherapy]. Wiener Medizinische Wochenschrift.

[CR5] Bhise V, Rajan SS, Sittig DF, Morgan RO, Chaudhary P, Singh H (2018). Defining and measuring diagnostic uncertainty in Medicine: a systematic review. Journal Of General Internal Medicine.

[CR6] Blaxter M (1978). Diagnosis as category and process: the case of alcoholism. Social Science & Medicine Part A: Medical Psychology & Medical Sociology.

[CR7] Bolboacă, S. D. 2019. Medical Diagnostic Tests: A Review of Test Anatomy, Phases, and Statistical Treatment of Data. *Comput Math Methods Med, 2019*, 1891569. 10.1155/2019/1891569. 10.1155/2019/1891569 PMC6558629 31275427

[CR8] Bossuyt PM (2011). The thin line between hope and hype in biomarker research. Jama.

[CR9] Brown, P. 1995. Naming and framing: the social construction of diagnosis and illness. Journal of Health and Social Behavior, 34–52. 7560848

[CR10] Car J, Sheikh A, Wicks P, Williams MS (2019). Beyond the hype of big data and artificial intelligence: building foundations for knowledge and wisdom. Bmc Medicine.

[CR11] Chiffi D, Zanotti R (2017). Fear of knowledge: clinical hypotheses in diagnostic and prognostic reasoning. Journal Of Evaluation In Clinical Practice.

[CR12] Cohen RH, Teal SB (2022). Medication for early pregnancy termination. Jama.

[CR13] Copeland DD (1977). Concepts of disease and diagnosis. Perspectives In Biology And Medicine.

[CR14] Copp, T., J. Jansen, J. Doust, B. W. Mol, A. Dokras, and K. McCaffery. 2017. Are expanding disease definitions unnecessarily labelling women with polycystic ovary syndrome? *BMJ, 358*, j3694. 10.1136/bmj.j3694. 10.1136/bmj.j3694 28814559

[CR15] Engelhardt, H. T. Jr. 1985. Typologies of disease: Nosologies revisited. Logic of Discovery and diagnosis in medicine, 56–71.

[CR16] Esserman LJ, Thompson IM, Reid B, Nelson P, Ransohoff DF, Welch HG, Srivastava S (2014). Addressing overdiagnosis and overtreatment in cancer: a prescription for change. The Lancet Oncology.

[CR17] Evans DW, Lucas N, Kerry R (2016). Time, space and form: necessary for causation in health, disease and intervention?. Medicine, Health Care And Philosophy.

[CR18] Fox S, Do T (2013). Getting real about Big Data: applying critical realism to analyse Big Data hype. International Journal of Managing Projects in Business.

[CR19] Frohlich H, Balling R, Beerenwinkel N, Kohlbacher O, Kumar S, Lengauer T, Zupan B (2018). From hype to reality: data science enabling personalized medicine. Bmc Medicine.

[CR20] Ginsburg, G. S., and H. F. Willard. 2009. *Essentials of genomic and personalized medicine*. Academic Press.

[CR21] Goldfield NI (2014). Big data—hype and promise. The Journal of ambulatory care management.

[CR22] Graber ML (2013). The incidence of diagnostic error in medicine. Bmj Quality & Safety.

[CR23] Graber ML, Franklin N, Gordon R (2005). Diagnostic error in internal medicine. Archives Of Internal Medicine.

[CR24] Han PK, Klein WM, Arora NK (2011). Varieties of uncertainty in health care: a conceptual taxonomy. Medical Decision Making.

[CR25] Ho D, Quake SR, McCabe ER, Chng WJ, Chow EK, Ding X, Ho C-M (2020). Enabling technologies for personalized and precision medicine. Trends Biotechnol.

[CR26] Hofmann B (2001). The technological invention of disease. Medical Humanities.

[CR27] Hofmann, B. 2002. On the triad disease, illness and sickness. *Journal of Medicine and Philosophy, 27*(6), 651–674. Retrieved from http://jmp.oxfordjournals.org/. 10.1076/jmep.27.6.651.13793 12607162

[CR28] Hofmann B (2017). Overdiagnostic uncertainty. European Journal of Epidemiology.

[CR29] Hofmann B (2017). Technological Invention of Disease. Encyclopedia of Creativity, Invention, Innovation and Entrepreneurship.

[CR32] Hofmann, B. M. 2019a. Back to Basics: Overdiagnosis Is About Unwarranted Diagnosis. *American Journal of Epidemiology, 188*(10), 1812–1817. doi:http://dx.doi.org10.1093/aje/kwz148. 10.1093/aje/kwz148 31237330

[CR33] Hofmann, B. M. 2019b. Biases and imperatives in handling medical technology. Health Policy and Technology, 8, 377–385. 10.1016/j.hlpt.2019.10.005.

[CR34] Hofmann, B. M. 2019c. Expanding disease and undermining the ethos of medicine. *European Journal of Epidemiology (EJE), 34*, 613–619. doi:http://dx.doi.org10.1007/s10654-019-00496-4. 10.1007/s10654-019-00496-4 30796581

[CR30] Hofmann, B. 2020. Progress bias versus status quo bias in the ethics of emerging science and technology. *Bioethics, 34*(3), 252–263. doi:http://dx.doi.org10.1111/bioe.12622. 10.1111/bioe.12622 31617222

[CR35] Hofmann, B. M., and K. B. Lysdahl. 2021. *Types of diagnostic uncertainty – defining them and addressing them*. Paper presented at the Philosophy of Advanced Medical Imaging, online. Workshop retrieved from.

[CR31] Hofmann B, Welch HG (2017). New diagnostic tests: more harm than good. Bmj.

[CR36] Irving G, Holden J (2013). The time-efficiency principle: time as the key diagnostic strategy in primary care. Family Practice.

[CR37] Jutel A (2011). Classification, disease, and diagnosis. Perspectives In Biology And Medicine.

[CR38] Kassirer, J. P. 1989. *Our stubborn quest for diagnostic certainty*. vol. 320. 1489–1491. Mass Medical Soc. In, . 10.1056/NEJM198906013202211 2497349

[CR39] Keers RY (1981). Laennec: his medical history. Thorax.

[CR40] Labrique A, Vasudevan L, Chang LW, Mehl G (2013). H_pe for mHealth: more “y” or “o” on the horizon?. Int J Med Inform.

[CR41] Leder D, Jacobson K (2014). Health and disease: the experience of health and illness. Encyclopedia of Bioethics.

[CR42] Liddell, H. G., H. S. Jones, and R. Scott. 2011. *The Online Liddell-Scott-Jones Greek-English lexicon*. University of California, Irvine.

[CR43] Mayerfeld, J. 1999. *Suffering and moral responsibility*. Oxford University Press on Demand.

[CR44] Mazzanti M, Shirka E, Gjergo H, Hasimi E (2018). Imaging, Health Record, and Artificial Intelligence: hype or hope?. Current Cardiology Reports.

[CR45] Mohanty, A., S. K. Mohanty, S. Rout, and C. Pani. 2021. *Liquid Biopsy, the hype vs. hope in molecular and clinical oncology* Paper presented at the Seminars in oncology. 10.1053/j.seminoncol.2021.06.002 34384614

[CR46] Moynihan R (2011). Medicalization. A new deal on disease definition. Bmj.

[CR47] Moynihan R (2013). What is disease? And why it’s a healthy question. Bmj.

[CR48] National Cancer Institute (2015). NCI dictionary of cancer terms. NCI Dict Cancer Terms.

[CR49] Nayarisseri A, Khandelwal R, Tanwar P, Madhavi M, Sharma D, Thakur G, Singh SK (2021). Artificial intelligence, big data and machine learning approaches in precision medicine & drug discovery. Current drug targets.

[CR50] Newman-Toker DE, Pronovost PJ (2009). Diagnostic errors–the next frontier for patient safety. Jama.

[CR51] Nickel B, Barratt A, Copp T, Moynihan R, McCaffery K (2017). Words do matter: a systematic review on how different terminology for the same condition influences management preferences. British Medical Journal Open.

[CR52] Norman GR, Eva KW (2010). Diagnostic error and clinical reasoning. Medical Education.

[CR53] Norman GR, Monteiro SD, Sherbino J, Ilgen JS, Schmidt HG, Mamede S (2017). The causes of errors in clinical reasoning: cognitive biases, knowledge deficits, and dual process thinking. Academic Medicine.

[CR54] Pinto A, Brunese L (2010). Spectrum of diagnostic errors in radiology. World journal of radiology.

[CR55] Risør, T. 2016. Where does a diagnosis come from: Questions about the local context in diagnostic reasoning. *2016, 13*(25). 10.7146/tfss.v13i25.24920.

[CR56] Rodríguez J, Avila J, Rolfo C, Ruíz-Patiño A, Russo A, Ricaurte L, Recondo G (2021). When tissue is an issue the liquid biopsy is nonissue: a review. Oncology and Therapy.

[CR57] Rogers WA, Mintzker Y (2016). Casting the net too wide on overdiagnosis: benefits, burdens and non-harmful disease. Journal Of Medical Ethics.

[CR58] Rogers WA, Walker MJ (2017). The line-drawing Problem in Disease Definition. Journal Of Medicine And Philosophy.

[CR59] Sadegh-Zadeh, K. 2012. Handbook of analytic philosophy of medicine.

[CR60] Santosh, K., and L. Gaur. 2021. AI in Precision Medicine. In *Artificial Intelligence and Machine Learning in Public Healthcare*, 41–47. Springer.

[CR61] Seely AJ (2013). Embracing the certainty of uncertainty: implications for health care and research. Perspectives in biology and medicine.

[CR62] Smith AK, White DB, Arnold RM (2013). Uncertainty: the other side of prognosis. New England Journal Of Medicine.

[CR63] Stempsey WE (1999). Disease and diagnosis: value-dependant realism.

[CR67] Töpfer F, Wiesing U (2005). The medical theory of Richard Koch I: theory of science and ethics. Medicine Health Care and Philosophy.

[CR64] Topol E (2012). The Creative Destruction of Medicine: how the Digital Revolution will create Better Health Care.

[CR65] Topol, E. 2019. *Deep medicine: how artificial intelligence can make healthcare human again*. Hachette UK.

[CR66] Tresker, S. 2020. A typology of clinical conditions. *Studies in History and Philosophy of Science Part C: Studies in History and Philosophy of Biological and Biomedical Sciences*, 101291. 10.1016/j.shpsc.2020.101291. 10.1016/j.shpsc.2020.101291 PMC7243781 32513474

[CR68] Vitor Pordeus M, Rosenberg L (2017). Disease as Oracle: Anamnesis, diagnosis and prognosis; past, Present and Future. EC Psychology and Psychiatry.

[CR69] Welch HG (2015). Less Medicine, more health: 7 assumptions that drive too much medical care.

[CR70] Welch HG, Schwartz L, Woloshin S (2011). Overdiagnosed: making people sick in the pursuit of health.

[CR71] Whitbeck C (1977). Causation in medicine: the disease entity model. Philosophy of Science.

[CR72] White, W. A. 1926. *The meaning of Disease: an Inquiry in the field of Medical Philosophy*. Williams & Wilkins.

[CR73] Whooley O, Barker KK (2021). Uncertain and under quarantine: toward a sociology of medical ignorance. Journal of Health and Social Behavior.

[CR74] Wieland, W. 2013. Diagnose. In *Diagnose*: de Gruyter.

[CR75] Wynne, B. 1980. Technology, risk and participation: On the social treatment of uncertainty. *Society, technology and risk assessment*, 173–208.

[CR76] Wynne B (1992). Uncertainty and environmental learning: reconceiving science and policy in the preventive paradigm. Global environmental change.

[CR77] Young, S. 2021. *The Science and Technology of growing Young: an Insider’s Guide to the breakthroughs that will dramatically extend our Lifespan… and what you can do right now*. BenBella Books.

[CR78] Yudkin JS, Montori VM (2014). The epidemic of pre-diabetes: the medicine and the politics. The BMJ.

